# Ultra processed food consumption and nutrients adequacy among cancer survivors in Lebanon

**DOI:** 10.1186/s12889-026-26547-6

**Published:** 2026-02-20

**Authors:** Remie El Helou, Bernard Srour, Ruba Hadla, Riwa Azar, Ziyad R. Mahfoud, Mathilde Touvier, Sally Temraz, Jana Jabbour

**Affiliations:** 1 Department of Nutrition, School of Arts and Sciences, Lebanese American University, Beirut, Lebanon; 2 Division of Human Nutrition and Health, Wageningen University and Research, Wageningen, Netherlands; 3 Nutritional Epidemiology Research Team (EREN), Université Sorbonne Paris Nord and Université Paris Cité, INSERM, INRAE, CNAM, Center of Research in Epidemiology and StatisticS (CRESS), Bobigny, France; 4 Nutrition And Cancer Research Network (NACRe Network), Jouy-en-Josas, France; 5 Department of Medical Center IT Applications, American University of Beirut, Beirut, Lebanon; 6 Department of Internal Medicine, American University of Beirut Medical Center, Beirut, Lebanon; 7 Department of Medical Education, Weill Cornell Medicine, Doha, Qatar

**Keywords:** Ultra processed food, Diet adequacy, Diet quality, Cancer survivors, Lebanon

## Abstract

Evidence on the impact of ultra-processed food (UPF) intake on diet quality among cancer survivors remains limited. This study examined UPF consumption and nutrient intake adequacy among cancer survivors in Lebanon.

In this cross-sectional study, adult cancer survivors in remission for at least three months were recruited from two medical centers. Dietary intake was assessed using a validated food frequency questionnaire (FFQ), and food items were categorized according to the NOVA classification. Nutrient adequacy ratio (NAR) and mean adequacy ratio (MAR) were calculated. Participant characteristics, nutrient adequacy, and macronutrient intakes were compared across UPF quartiles using Chi-square tests, and ANOVA. Logistic regression was performed to identify predictors of nutrient inadequacy.

The study included 268 participants (mean age: 59 years; 83% female). UPF accounted for 8 ± 7% of total food weight and 17.5 ± 11% of energy intake. Most participants did not meet requirements for potassium (95%), vitamin A (87%), and vitamin D (99%) with mean intakes of 2,527 ± 1236 mg, 350 ± 175 Retinol Activity Equivalents, and 0.8 ± 1.2 µg, respectively. Higher UPF intake was significantly associated with higher energy (*p* < 0.001), carbohydrate (*p* < 0.001), protein (*p* = 0.017), fat (*p* < 0.001), and saturated fat intake (*p* < 0.001), and with lower vitamin C adequacy (*p* = 0.02). In multivariable analysis, higher education predicted lower odds of nutrient inadequacy (AOR = 0.26, 95% CI: 0.1–0.65, *p* = 0.004), while UPF intake did not.

Despite the relatively low UPF contribution, significant micronutrient inadequacies were observed among cancer survivors. These findings underscore the importance of integrating dietary counseling into cancer care to address nutrient gaps and promote healthier food choices.

## Novelty and impact

This is the first study in Lebanon and the MENA region to assess ultra-processed food (UPF) consumption and nutrient adequacy among cancer survivors. Although UPF contribution was relatively low, significant micronutrient inadequacies were observed. The findings underscore the importance of integrating nutrition counseling into cancer care and advancing public health policies to reduce UPF availability while promoting nutrient-rich, whole foods

## Background

Cancer poses a heavy economic and public health burden in Lebanon, exceeding that of many other countries in the Middle East and North Africa (MENA) region [1, 2]. Over the past decade, this burden has steadily increased [3], making cancer the third leading contributor to disability-adjusted life-years and accounting for 8% of the country’s total disease burden [2]. Despite advances in diagnosis and treatment, cancer survivors remain at elevated risk of functional decline, recurrence, and non-communicable diseases (NCDs) compared with healthy peers [4, 5]. Amid ongoing shortages of cancer medications and limited access to care in Lebanon [6], comprehensive cancer management emphasizing prevention, early detection, and efficient therapeutic strategies is urgently needed [7].

Nutrient adequacy, defined as sufficient intake of essential nutrients to meet physiological needs [8] has been linked to a lower risk of cancer recurrence and improved survival [9]. Consuming a diet rich in vitamins, minerals, fiber, and healthy fats supports cancer survivors in managing treatment-related side effects, preventing malnutrition and sarcopenia, enhancing recovery, and improving overall quality of life [10, 11]. Micronutrients, in particular, are critical for immune function, cellular repair, and modulation of inflammation in this population [12].

Ultra-processed food (UPF), as defined by the NOVA classification system, results from extensive industrial processing, such as fractioning, hydrolysis, chemical modifications, and industrial use of some ingredients such as “cosmetic additives” [13, 14], and its consumption has been linked to nutrient inadequacy. A meta-analysis indicated that in the general population, increased consumption of UPFs is associated with lower intakes of fiber, protein, potassium, zinc, magnesium, and vitamins A, C, D, E, B12, and niacin, and an elevated consumption of added sugars, unhealthy fats, and sodium [15]. Moreover, UPF consumption is suspected to be associated with compromised overall health and well-being, and increased risk of cancer [16, 17]. A large prospective cohort study including around 100,000 participants from the French NutriNet-Santé cohort showed that a 10% increase in UPF consumption was related to a more than 10% rise in overall and breast cancer risk [18]. A systematic review and meta-analysis of studies assessing UPF consumption and cancer risk have shown a consistent significant association between intake of UPF and the risk of overall and several cancer types such as colorectal, breast, and pancreatic cancer [19]. These data suggest that it might be strategic to assess and limit the consumption of UPF to reduce the burden related to cancer [16].

In Lebanon, UPF contributes an estimated 25–47% of total energy intake in the general population [20, 21], yet no comparable data exist for cancer survivors. Evidence on nutrient adequacy among cancer survivors is also limited in Lebanon and the broader MENA region [22]. To our knowledge, this is the first study in Lebanon to examine UPF consumption among cancer survivors, their nutrient intake adequacy, and the association between these factors.

## Methods

This is a cross-sectional study conducted between August 2018 and February 2022 at the American University of Beirut Medical Center (AUBMC) and Makassed General Hospital (MGH) in Beirut, Lebanon. It is part of a larger parent study, the full methodology of which has been previously published [23]. Reporting of the study methodology and findings is based on the Strengthening the Reporting of Observational Studies in Epidemiology guidelines [24] and the study followed the ethical principles outlined in the Declaration of Helsinki and the Belmont report. Approval for the research project was obtained from the Institutional Review Board (IRB) at the American University of Beirut (protocol number SBS-2018-0168) and the ethics committee at MGH.

Patients attending private clinics and dispensaries at AUBMC and MGH were approached by research team members, who provided detailed information about the study’s objectives, potential benefits, and risks involved. Interested patients who met the inclusion criteria and were willing to participate provided both written and oral consent [23]. Eligible participants were adults (≥ 18 years) previously diagnosed with one of the eight most common cancers in Lebanon, according to the National Cancer Registry (breast, respiratory, colorectal, prostate, lymphoma [Hodgkin and non-Hodgkin], bladder, stomach, and leukemia), and in remission for ≥ 3 months [25]. Remission status was confirmed through the most recent CT report and/or oncologist notes in medical records. Patients with terminal illness, receiving palliative care, or with visual/cognitive impairments were excluded.

### Assessment tools

Participants provided information on lifestyle factors including education, socioeconomic status, occupation, and living arrangements. Household crowding was assessed using the Crowding Index (CI), calculated as the number of residents divided by the number of rooms (excluding bathrooms, balconies, and kitchens); CI > 1 indicated crowding [26]. Food insecurity was evaluated using the culturally validated Arab Family Food Security Scale [27], with scores ≤ 2 indicating food security and ≥ 3 indicating food insecurity. Physical activity was assessed using the Arabic version of the International Physical Activity Questionnaire–Short Form (IPAQ-SF) [28], and participants were classified as having low, moderate, or high activity levels [28].

Dietary intake was collected through a 119-item Food Frequency Questionnaire (FFQ) adapted from a validated Lebanese version [29]. Participants reported frequency and portion sizes of commonly consumed foods, with household measures used to aid portion size estimation. Nutrient analysis was performed in Microsoft Excel (Microsoft Corporation, Redmond, WA, USA) using food composition data from local sources [30–32] and the United States Department of Agriculture FoodData Central [33]. Supplement use (vitamins, minerals, or other dietary products) was also recorded.

UPF consumption was determined using the NOVA classification system [34]. Each FFQ item was assigned a NOVA score ranging from 1 to 4 (1 = minimally processed, 2 = processed culinary ingredients, 3 = processed foods, 4 = UPF) [35] by two graduate nutrition students and two licensed dietitians independently. Disagreements were resolved by a senior dietitian. UPF intake was expressed as percent of total food weight derived from UPFs, rather than energy contribution to capture items with negligible calories but high processing (e.g., artificially sweetened beverages) [36, 37].

Micronutrient adequacy was evaluated using the Nutrient Adequacy Ratio (NAR) and Mean Adequacy Ratio (MAR), comparing individual intakes with age- and sex-specific Estimated Average Requirements (EAR) or Adequate Intakes (AI) [38, 39]. For sodium and potassium, AI values were used due to the absence of EARs. MAR was calculated as the mean of truncated NAR values (≤ 1) across assessed micronutrients [39]. A MAR < 0.75 indicated overall nutrient inadequacy [39–41].

### Sample size and statistical considerations

The present study is part of a larger parent study, for which the original sample size calculation was based on adherence of cancer survivors to dietary and physical activity guidelines [23]. A post-hoc power analysis was subsequently performed for UPF consumption. The analysis used data from the UK Biobank cohort, where median UPF consumption (as % of food weight) was 29.3% (IQR: 19.5–40.6) in males and 25.8% (IQR: 16.6–36.4) in females [42]. Assuming 95% confidence (Z = 1.96), a margin of error (MOE) of 2, and standard deviation estimated as IQR/1.35, the required minimum sample size was 222, calculated using the formula (Z²σ²/MOE²).

Baseline characteristics and micronutrient adequacy were compared across UPF intake quartiles using Chi-square tests for categorical variables and one-way ANOVA for continuous variables. Energy (kcal/day), fiber (g/day), and macronutrients (g/day) were also compared across UPF quartiles using ANOVA. Fatty acids [total fat, saturated fatty acids (SFA), monounsaturated fatty acids (MUFA), polyunsaturated fatty acids (PUFA), and trans fatty acids (TFA)] were expressed as percent of total energy intake and compared across quartiles of UPF as well. To identify the main contributors to UPF intake, the top 10 UPF items were determined for each participant, then aggregated to generate a ranked list for the sample. Predictors of reduced diet adequacy (MAR < 0.75 vs. ≥0.75) were examined using univariable and multivariable logistic regression models (enter method). Variables with *p* ≤ 0.20 in univariable models were entered into multivariable analyses. Age, gender, and UPF intake were forced into the multivariable model for their clinical relevance. Multicollinearity was defined as correlation coefficients > 0.7 or variance inflation factors > 10 [43]. Statistical significance was reported at the conventional level of *p* < 0.05. Since data was not missing neither for the outcomes of interest nor for the variables entered in the regression model, data imputation was not performed. Data management and analysis were performed on IBM SPSS Statistics version 29 (SPSS Inc., Chicago, IL, USA).

## Results

The sample included 268 cancer survivors with a mean age of 59 ± 23 years; 83% were female, 67% have overweight or obesity, and approximately half resided in urban areas and held a university degree. Supplement use was reported by 72% of participants, predominantly vitamin D (58%), calcium (31%), and multivitamins (8%). Only 10% were highly active, 25% exercise moderately, and 65% have low physical activity levels. Food insecurity was reported by the majority (72%) (Table 1). Mean UPF intake was 8 ± 7% of total food weight and 17.5 ± 11% of total energy intake. Baseline characteristics were compared across quartiles of UPF intake (by percent weight), with no statistically significant differences observed (Table 1). The top contributors to UPF consumption among participants were assessed by food weight and are presented in Table 2. Western sweets (cupcakes, muffins, cakes, doughnuts, and chocolate) ranked first, accounting for 8.7% of UPF intake, followed by potato chips (6.9%), spreadable cheeses (4.8%), ice creams, and biscuits (4.3%) (Table 2).

**Table 1:** Subjects’ baseline characteristics by quartiles of ultra processed food consumption calculated as percent food weight

Characteristic		Overall (*n* = 268)	Quartiles of UPF intake by weight				*P* value
			Q1 (*n* = 70)	Q2 (*n* = 64)	Q3 (*n* = 68)	Q4 (*n* = 66)	
Age, mean (SD)		59 (23)	57 (12)	60 (12)	57 (13)	55 (12)	0.119
Female, n (%)		222 (83)	56 (80)	52 (81)	59 (87)	55 (83)	0.739
BMI (Kg/m²), n (%)	Underweight	2 (0.8)	0 (0)	0 (0)	1 (2)	1 (2)	0.403
	Normal	79 (33)	22 (36)	19 (33)	18 (29)	20 (32)	
	Overweight	87 (36)	20 (33)	16 (28)	30 (48)	21 (34)	
	Obese	75 (31)	19 (31)	22 (39)	14 (22)	20 (32)	
Living alone, n (%)	Living alone	18 (7)	4 (6)	2 (3)	7 (10)	5 (8)	0.408
	Living with others	250 (93)	66 (94)	62 (97)	61 (90)	61 (92)	
Living area, n (%)	urban/city	157 (59)	44 (63)	35 (55)	40 (59)	38 (58)	0.442
	suburban	65 (24)	12 (17)	19 (30)	20 (29)	14 (21)	
	rural	46 (17)	14 (20)	10 (16)	8 (12)	14 (21)	
CI, n (%)	< 1	134 (50)	31 (45)	37 (58)	38 (56)	28 (43)	0.217
	> 1	132 (50)	38 (55)	27 (42)	30 (44)	37 (57)	
Education, n (%)	Primary school or less	51 (19)	18 (26)	17 (27)	9 (13)	7 (11)	0.107
	High School	84 (31)	17 (25)	21 (33)	22 (32)	24 (36)	
	University	132 (49)	34 (49)	26 (41)	37 (54)	35 (53)	
Working status, n (%)	Work and/or Study	110 (41)	31 (44)	22 (34)	26 (38)	31 (47)	0.447
	Stay at home	158 (59)	39 (56)	42 (66)	42 (62)	35 (53)	
Smoking, n (%)	Current smoker	78 (30)	17 (24)	19 (30)	21 (32)	21 (33)	0.941
	Ex-smoker	38 (15)	11 (16)	8 (13)	10 (15)	9 (14)	
	Non-smoker	146 (56)	42 (60)	36 (57)	34 (52)	34 (53)	
Supplement/vitamin intake, n(%)		190 (72)	48 (70)	49 (78)	49 (73)	44 (68)	0.596
PA level, n (%)	Low	174 (65)	42 (60)	44 (69)	43 (63)	45 (68)	0.784
	Moderate	66 (25)	18 (26)	14 (22)	17 (25)	17 (26)	
	High	28 (10)	10 (14)	6 (9)	8 (12)	4 (6)	
Provision of dietary guidelines during survivorship, n (%)		62 (23)	16 (23)	12 (19)	17 (25)	17 (26)	0.808
Primary cancer, n (%)	Breast	187 (70)	47 (67)	46 (72)	53 (79)	41 (62)	0.356
	Hematology	40 (15)	10 (14)	11 (17)	7 (10)	12 (18)	
	GI	22 (8)	7 (10)	3 (5)	3 (5)	9 (14)	
	Respiratory	7 (3)	4 (6)	0 (0)	2 (3)	1 (2)	
	Others	11 (4.1)	2 (2.9)	4 (6.3)	2 (3)	3 (5)	
Charlson Comorbidity Index, mean (SD)		1.7 (1.3)	1.64 (1.3)	2 (1.4)	1.7 (1.2)	1.4 (1)	0.106
Quality of life, n (%)	High QoL	112 (42)	33 (47)	24 (38)	28 (41)	27 (41)	0.720
	Low QoL	156 (58)	37 (53)	40 (63)	40 (59)	39 (59)	
Food security, n (%)	Food Secure	75 (28)	16 (23)	15 (23)	20 (29)	24 (36)	0.268
	Food Insecure	193 (72)	54 (77)	49 (77)	48 (71)	42 (64)	
UPF consumption, % food weight, mean (SD)		8 (7)					
UPF consumption, % of total energy intake, mean (SD)		17.5 (11)					

Q1: quartile 1, Q2: quartile 2, Q3: quartile 3, Q4: quartile 4

UPF: Ultra Processed Food; BMI: body mass index; CI: crowding index; PA: physical activity; QoL: quality of life

**Table 2:** Top ten food items contributing to ultra processed food intake

Rank	Food item	Contribution (% weight of food consumed)
1	Western Sweets (Cupcakes, muffins, cakes, doughnuts and chocolate)	8.7
2	Potato chips, regular	6.9
3	Cheeses (low fat and regular)	4.8
4	Ice cream and popsicle	4.3
5	Biscuits	1.9
6	Arabic sweets	1.5
7	Puddings	1.5
8	Kaak & croissants	1.3
9	Candies	1.1
10	Vegetable ghee	1.0

Table 3 presents macronutrients and fiber intake across quartiles of UPF consumption. The overall mean daily energy intake was 2086 ± 972 kcal, with participants in Q4 reporting significantly higher intake (2624 ± 1346 kcal) compared with lower quartiles (*p* < 0.001). Carbohydrate intake showed a similar pattern, with an overall mean of 262 ± 122 g/day, peaking at 318 ± 178 g/day in Q4 (*p* < 0.001). Protein intake also increased significantly across UPF quartiles, with an overall mean of 79 ± 34 g/day and a maximum of 90 ± 43 g/day in Q4 (*p* = 0.017). Mean fat intake differed significantly between quartiles (*p* < 0.001), with Q4 participants reporting the highest intake (136 ± 76 g/day) compared with Q1 (89 ± 62 g/day). Fiber intake showed no significant differences across quartiles, with an overall mean of 25 ± 14 g/day. The percent of energy coming from fat differed significantly across quartiles (*p* = 0.034), ranging from 42 ± 9% in Q1 to 48 ± 20% in Q4. MUFA and PUFA intake did not differ significantly across quartiles, with overall intakes of 13% and 6%, respectively, falling within recommended ranges [44]. In contrast, SFA intake averaged 16 ± 4% and varied significantly across quartiles (*p* < 0.001), ranging from 13 ± 3% in Q1 to 19 ± 4% in Q4. TFA intake showed no significant differences (*p* = 0.89), with an overall mean of 0.24% (Table 3).

**Table 3:** Macronutrients and fiber intake per quartiles of ultra processed food consumption calculated as percent food weight

Nutrient	Overall, mean (SD)	UPF Quartiles, mean (SD)				*P* value
		Q1 (*n* = 70)	Q2 (*n* = 64)	Q3 (*n* = 68)	Q4 (*n* = 66)	
Energy (Kcal/day)	2086 (972)	1876 (893) ͣ	1932 (562) ͣ	1924 (712) ͣ	2624 (1346)ꙺ	< 0.001
Carbohydrates (grams)	262 (122)	247 (102) ͣ	253 (84) ͣ	233 (91) ͣ	318 (178) ꙺ	< 0.001
Protein (grams)	79 (34)	74 (34) ͣ	75 (22) ͣ	77 (31) ͣ	90 (43) ꙺ	0.017
Fat (grams)	103 (57)	89 (62) ͣ	95 (31) ͣ	94 (34) ͣ	136 (76) ꙺ	< 0.001
Fibers (grams/day)	25 (14)	28 (18)	26 (11)	22 (10)	24 (13)	0.065
Percent Energy intake (%)						
Fat	44 (12)	42 (9) ͣ	44 (7) ͣ	45 (7) ͣ	48 (20) ꙺ	0.034
MUFA	13 (12)	13 (5)	14 (5)	13 (5)	13 (7)	0.850
PUFA	6 (6)	5 (2)	6 (2)	5 (2)	7 (12)	0.580
SFA	16 (4)	13 (3)ᵃ	15 (3)ᵇ	16 (3) ᵇ	19 (4)ᶜ	< 0.001
TFA	0.24 (0.2)	0.25 (0.2)	0.22 (0.3)	0.25 (0.27)	0.23 (0.15)	0.890

Q1: quartile 1, Q2: quartile 2, Q3: quartile 3, Q4: quartile 4

UPF: Ultra Processed Food; MUFA: monounsaturated fatty acids; PUFA: polyunsaturated fatty acids; SFA: saturated fatty acids; TFA: trans fatty acids

ᵃᵇᶜgroups with a letter in common are not statistically significant and those with no letter in common are statistically significant

Table 4 presents micronutrients intake inadequacy across quartiles of UPF consumption. The nutrient intake was not adjusted to vitamin/supplement intake. The mean MAR for the overall sample was 0.82 ± 0.08. Most participants failed to meet their individual requirements for potassium (95%), vitamin A (87%), and vitamin D (99%), with mean intakes of 2527 ± 1236 mg, 350 ± 175 RAE, and 0.8 ± 1.2 µg, respectively. Significant differences in inadequacy rates across UPF quartiles were observed for calcium, potassium, thiamin, vitamin C, and folate. For calcium, half of the participants reported inadequate intake, with the proportion decreasing significantly across quartiles: 63% in Q1 versus 36% in Q4 (*p* = 0.01). Potassium inadequacy reached 100% in Q3, compared with 91% in Q4 (*p* < 0.01). Thiamin inadequacy was highest in Q3 (47%), whereas lower rates were observed in Q1 (26%) and Q2 (27%) (*p* = 0.02) (Table 4).

**Table 4:** Micronutrients intake inadequacy by quartiles of ultra processed food calculated as percent food weight

Nutrient	Overall, *n*(%)	Q1, *n*(%)	Q2, *n*(%)	Q3, *n*(%)	Q4, *n*(%)	*P* value
Sodium	33 (12)	13 (19)	6 (9)	9 (13)	5 (8)	0.210
Calcium	130 (49)	44 (63) ͣ	33 (52)	29 (43)	24 (36) ͣ	0.013
Phosphorus	15 (6)	7 (10)	2 (3)	4 (6)	2 (3)	0.300
Potassium	254 (95)	66 (94)	62 (97)	68 (100) ͣ	58 (88) ͣ	0.009
Iron	19 (7)	5 (7)	2 (3)	7 (10)	5 (8)	0.460
Thiamin	91 (34)	18 (26)	17 (27)	32 (47) ͣ	24 (36)	0.020
Riboflavin	46 (17)	18 (26)	9 (14)	11 (16)	8 (12)	0.150
Niacin	127 (47)	35 (50)	29 (45)	37 (54)	26 (39)	0.340
Vitamin C	42 (16)	8 (11)^a, b,c^	4 (6) ͣ	17 (25)^b^	13 (20)^c^	0.010
Vitamin A	233 (87)	63 (90)	54 (84)	62 (91)	54 (82)	0.300
Vitamin D	267 (99)	70 (100)	64 (100)	68 (100)	65 (98)	0.480
Zinc	71 (26.5)	18 (26)	17 (27)	18 (27)	18 (27)	0.900
Folate	71 (26.5)	16 (23)	11 (17)	24 (35)	20 (30)	0.090

Q1: quartile 1, Q2: quartile 2, Q3: quartile 3, Q4: quartile 4

ͣ Significantly different groups

ᵃᵇᶜgroups with a letter in common are not statistically significant and those with no letter in common are statistically significant

Micronutrient intake adequacy was evaluated using the Nutrient Adequacy Ratio (NAR) and the Mean Adequacy Ratio (MAR). NAR was calculated by comparing each micronutrient intake to the respective age and sex specific EAR or AI values, AI was used for sodium and potassium. MAR, an index for overall nutrient adequacy, was calculated by averaging the NAR values, with MAR < 0.75 indicating nutrient inadequacy

Table 5 presents the multivariable regression analysis of predictors of nutrient inadequacy. In both univariable and multivariable models, educational level was the only factor significantly associated with nutrient inadequacy. Compared with participants with primary education or less, those with a high school education had significantly lower odds of inadequacy (AOR = 0.26, 95% CI: 0.10–0.65, *p* = 0.004), while participants with a university education showed even lower odds (AOR = 0.12, 95% CI: 0.04–0.30, *p* < 0.001). UPF intake was not a significant predictor of nutrient adequacy in either crude or adjusted models. However, in the multivariable analysis, participants in the third and fourth quartiles of UPF intake had higher odds of inadequacy (AOR = 2.07 and 1.96, respectively) compared with those in the first quartile, although these associations did not reach statistical significance (Table 5).

**Table 5:** Univariable and multivariable logistic regression models of the predictors of nutrient inadequacy

Variable		Unadjusted			Adjusted		
		OR	95% CI	*P* value	OR	95% CI	*P* value
Age		1.01	0.98–1.04	0.4	0.99	0.96–1.02	0.686
Gender							
	Male (ref)	1	-	-	1	-	-
	Female	1.4	0.5–3.9	0.48	1.3	0.44–3.9	0.628
Educational level							
	Primary school or below (ref)	1	-	-	1	-	-
	High school	0.3	0.12–0.7	0.006	0.26	0.09–0.65	0.005
	University	0.2	0.07–0.4	< 0.001	0.11	0.04–0.31	< 0.001
Smoking							
	Current smoker (ref)	1	-	-	1	-	-
	Ex- smoker	2.7	0.9–8.1	0.07	2.57	0.76–8.65	0.126
	Non-smoker	1.9	0.7–4.6	0.2	2.47	0.94–6.5	0.066
Crowding index							
	< 1 (ref)	1	-	-	-	-	-
	> 1	1. 3	0.6–2.6	0.45	-	-	-
Physical activity level							
	Low (ref)	1	-	-	-	-	-
	Moderate	0.7	0.3–1.6	0.44	-	-	-
	High	0.4	0.09–1.78	0.23	-	-	-
Charlson comorbidity index		1.17	0.9–1.5	0.22	-	-	-
Food security							
	Food insecure (ref)	1	-	0.29	-	-	-
	Food secure	1.5	0.7–3.5	0.3	-	-	-
Quartiles of UPF intake							
	Q1 (ref)	1	-	-	1	-	-
	Q2	1.1	0.4–3.15	0.85	0.54	0.16–1.75	0.306
	Q3	1.8	0.7–4.7	0.2	2.07	0.73–5.85	0.169
	Q4	1.2	0.4–3.4	0.7	1.96	0.7–5.46	0.199

Ref: reference group; Nutrient inadequacy based on mean adequacy ratio (MAR) < 0.75

UPF: Ultra Processed Food

Q1: quartile 1, Q2: quartile 2, Q3: quartile 3, Q4: quartile 4

Variables with *p* ≤ 0.20 in univariable models were entered into multivariable analyses, the rest were excluded

## Discussion

This study is the first in Lebanon and the MENA region to assess nutrient adequacy and UPF consumption among cancer survivors. The contribution of UPFs to total food weight was low (8%), yet inadequacies in key micronutrients, particularly potassium, vitamins A, and D, were noted. Macronutrient intakes varied across quartiles of UPF consumption, with participants in the highest quartile reporting significantly greater energy, carbohydrate, protein, and fat intakes, alongside higher SFA consumption. Notably, calcium and potassium inadequacies were most prevalent in Q1, while vitamin C and thiamin inadequacies were most frequent in Q3 and Q4, respectively.

### UPF consumption among cancer survivors

The mean UPF contribution in this cohort was lower than reports from cancer survivors in the UK Biobank (25–29% of total food weight) [42] and from the general Lebanese population, where UPFs contribute 25–47% of total energy intake [20, 21, 45]. Studies from European countries also reported higher UPF contribution as percent food weight, namely 17% in France, and 30% in the United Kingdom [46, 47]. The comparatively lower intakes may reflect a post-diagnosis shift toward healthier behaviors, consistent with evidence that cancer survivors often adopt more health-conscious dietary practices [48]. This aligns with the parent study of the current project which has demonstrated high adherence to American Cancer Society/American Institute for Cancer Research guidelines limiting alcohol, red meat, and energy-dense foods [23].

The main contributors to UPFs were Western-style sweets, potato chips, ice cream, and spreadable cheeses, underscoring the influence of Westernization and globalization on traditional Mediterranean diets which typically excludes UPF [49]. The shift in dietary patterns has led to a preference for convenient and palatable yet nutritionally poor food options among Lebanese individuals, including cancer survivors. Our findings are in line with international studies showing positive associations between UPF intake and higher energy and fat consumption, with SFA intake often exceeding recommended thresholds [50, 51]. Analyses of dietary data in an Italian cohort showed significantly higher intakes of energy and fat in the top UPF quartile [52]. Similar trends were observed for carbohydrate and protein intakes, although the differences across UPF quartiles were not significant [52]. Assessments of UPF intake in Brazil and Canada revealed higher energy and macronutrient intakes to be associated with increased UPF consumption [53, 54]. Studies focusing on fatty acid intakes have reported excessive consumption of total fat and SFA among individuals with higher UPF intake [50, 51], exceeding the recommended values [51].

### UPF intake and nutrient adequacy

In the adjusted regression model of our analysis, higher UPF quartiles were associated with increased odds of inadequacy, however these associations did not reach statistical significance, potentially due to limited power. A larger sample, estimated at around 400 participants, would be required to robustly examine this outcome. This finding could also be explained by the fact that supplement intake was not accounted for in the analysis, its use, particularly among individuals with higher socioeconomic or educational status, may have attenuated associations between UPF intake and nutrient adequacy. The low overall UPF intake among the survivors could also have affected the association between nutrient adequacy and UPF intake.

Higher UPF quartiles were associated with lower inadequacy of calcium and potassium intake. This contrasts with prior studies linking UPFs to reduced micronutrient quality [55]. In our cohort, this may be explained by dairy-based UPFs such as cheese and ice cream, which were among the key contributors to UPF intake and are relatively rich in these nutrients (e.g. western sweets, cheeses and ice cream). Conversely, vitamin C inadequacy was more prevalent at higher UPF intakes which is in line with Portuguese data showing worsening vitamin C adequacy with increased UPF consumption [56]. Educational level emerged as a predictor of nutrient inadequacy, with higher education associated with significantly lower odds of inadequacy. This finding has been consistently observed across diverse cancer survivor cohorts in Finland, Korea, and Canada [57–59], likely reflecting greater health literacy, better access to healthy foods, and stronger adherence to dietary recommendations.

### Public health implications

Growing evidence links higher UPF consumption to adverse long-term health outcomes [60]. Prospective cohort studies and meta-analyses have consistently shown that greater UPF intake is associated with increased all-cause mortality [60], as well as higher risk of overall and site-specific cancers, including colorectal and breast cancer [19]. These associations suggest that the health implications of UPF consumption extend beyond nutrient inadequacy and underscore the importance of dietary patterns that limit UPFs to reduce long-term disease burden in our region.

Educational attainment was a significant predictor of UPF consumption in our study. Research from the United Arab Emirates demonstrated a positive association between education and health literacy, with adequate health literacy nearly twice as prevalent among individuals with higher educational attainment [61]. Taken together, these findings suggest that education may influence UPF consumption through its effect on health literacy. Accordingly, we recommend the implementation of coordinated population-level interventions that address educational disparities and strengthen health and nutrition literacy. Aligning with the worldwide and Eastern Mediterranean Region (EMR) World Health Organization (WHO) guidance, we recommend strategies that incorporate structured nutrition counseling and strengthen food selection skills [62, 63]. These approaches aim to improve individuals’ ability to interpret nutrition labels, distinguish between minimally processed and ultra-processed foods, and make informed food choices that support adequate nutrient intake. At the population level, additional measures such as food fortification and clear front-of-pack nutrition labeling may further support informed and healthier food choices [64–67]. Complementary food-environment strategies, consistent with WHO Best Buys, should prioritize regulatory actions such as restricting the marketing of unhealthy foods, particularly to vulnerable populations, and improving the overall availability of healthier foods [68]. Although several countries in the EMR have advanced the implementation of these frameworks, Lebanon continues to lag behind, highlighting an urgent need for comprehensive public health reforms to protect survivors and the broader population from similar nutritional risks [67, 68].

### Strengths and limitations

The findings of the present study should be interpreted considering some limitations such as the cross-sectional design, reliance on FFQ self-reporting subject to recall bias, and a sample size insufficient to fully examine the UPF–nutrient adequacy relationship. Moreover, this study relied on self-reported dietary data and did not include validation against objective nutritional biomarkers, which may affect the precision of nutrient adequacy estimates. Additionally, while the NOVA classification is conducted systematically, the judgment for certain food items such as industrial breads and processed cheeses may be subject to ambiguity, despite expert reviewing it could introduce potential for misclassification. These findings, however, provide a valuable foundation for future longitudinal and intervention studies. Key strengths of this study include being the first regional assessment of UPF intake and nutrient adequacy among cancer survivors, the use of validated and culturally adapted tools, and rigorous classification of UPFs.

## Conclusion

The present study provides an examination of nutrient adequacy and UPF consumption among cancer survivors in Lebanon. Despite the relatively low contribution of UPFs to the diet, significant variations in macronutrient intakes and inadequacies in essential micronutrients were observed across UPF quartiles. Our findings highlight the need for targeted public health interventions at the individual and community levels. Strengthening dietary guidelines, promoting public awareness, and integrating nutrition counseling into cancer care are critical steps to mitigate the negative impacts of malnutrition during survivorship. Additionally, governmental policies should focus on reducing UPF availability and marketing while promoting access to whole and fresh foods, in addition to limiting access to foods high in sugar, salt, and fats. Future longitudinal and interventional studies are warranted to further clarify the relationship between UPF intake, nutrient adequacy, and clinical outcomes in this vulnerable population.

## Data Availability

The data that support the findings of this study are available upon request from the corresponding author. The data is not publicly available due to privacy and ethical restrictions.

## Abbreviations

AI: Adequate Intake
AOR: Adjusted Odds Ratio
AUBMC: American University of Beirut Medical Center
CI: Crowding Index
EAR: Estimated Average Requirement
FFQ: Food Frequency Questionnaire
IPAQ-SF: International Physical Activity Questionnaire–Short Form
IRB: Institutional Review Board
MAR: Mean Adequacy Ratio
MGH: Makassed General Hospital
MENA: Middle East and North Africa
MOE: Margin of Error
MUFA: Monounsaturated Fatty Acids
NAR: Nutrient Adequacy Ratio
NCDs: Non-Communicable Diseases
PUFA: Polyunsaturated Fatty Acids
SFA: Saturated Fatty Acids
SPSS: Statistical Package for Social Sciences
TFA: Trans Fatty Acids
UPF: Ultra-Processed Food
